# Supplementary material for: Determinants of anemia level among reproductive-age women in 29 Sub-Saharan African countries: A multilevel mixed-effects modelling with ordered logistic regression analysis
Source: PLoS One. 2023 Nov 29;18(11):e0294992. doi: 10.1371/journal.pone.0294992 (PMC10686498; doi:10.1371/journal.pone.0294992)
Supplement: Attachment — Submitted filename: Comments to PLOS ONE.docx [file pone.0294992.s001.docx]

Comments

I thank you for the opportunity to read and revise the manuscript “Determinants of anemia level among reproductive-age women in Sub-Saharan African countries: a multilevel mixed-effects analysis with ordered logistic regression modeling”.

The manuscript sounds very interesting and the promising data can significantly add information on the anemia level and influencing factors in SSA. However, some parts are very hard to read, and as well as there are a consistent number of statistical issues that need to be considered.

REQUESTED REVISIONS:

Abstract

1. In method section “Data from 205,627 reproductive-age women from the recent demographic and health survey of ….” Which DHS conducted year you used please specify.
2. Methods used should be reflected in the abstract very well and precisely.
3. Result part needs re-write and revision.

Introduction

1. Clear gap should be present other than methodological gap.
2. In general the background needs extensive revision.

Methods

1. Please use sub-heading as “data extraction method”, I haven’t see data extraction method and where it was obtained.
2. Line 113 “…. After managing missing observations” which missing mechanism technique you apply in this study. Please use “missing mechanism “as sub-heading explains more how you handle you missing data in your study.
3. Data cleaning procedure was not mention
4. For the whole method section, please try to reflect the real activities undertaken by organizations who conducted the method and data collection.
5. The other serious problem, that requires serious attention, I have seen in this section is that, the authors did fall short of accounting for the complexity of the DHS data. The data were collected via multi-stage sampling approach with clustering and stratification. So, this data should be analyzed with this sampling process in mind. See the DHS analysis manual for better understanding. I would argue that findings without accounting for the complexity of the data are erroneous and biased towards oversampled areas and settings, and would not reflect the situation of each country.
6. For each category of outcome variable cut point was not mention i.e. for not anemic, mild anemic, moderate anemic and sever anemic and cite where the cut point you found.
7. I haven’t seen any inclusion and exclusion criteria for this study.

Data management and statistical analysis

1. How you judge the variable to have a statistically significant association with anemia level? And how you link the strength ….. Please write in this section.
2. For the logistic regression model, you are going to use the Generalized Variance Inflation Factor (GVIF) since this model belongs to the class of generalized linear models. Results on GVIF are not presented.

Model building and selection

1. The model parameter estimation technique was not mentioned
2. What is your alternative model if the proportional odds model is failed…. I haven’t seen any other alternative ordinal model if the proportional odds model is failed….. I recommend the researcher to write other ordinal models (i.e. generalized ordered models, partial proportional odds model …) and when it applies these models in the method section.
3. Is deviance the model selection criteria??? The model selection criteria are based on AIC and BIC values but I haven’t seen in this section. In addition testing overall model fit is not mention.
4. I recommend including “marginal effect” i.e. the effect of each response category across the country.

Result

1. I think prevalence of anemia among reproductive-age woman should present for each country.
2. Result interpretation needs revision.
3. I think for this study country variation (substantial heterogeneity across each country) should reflect other than pooled prevalence.

Discussion

1. Some parts in the discussion are difficult to understand. Please carefully rewrite this section considering every single set of covariates reporting both significant and non-significant results.
2. Go through the whole document and edit technical issues

Conclusion

1. Your conclusion should be revised and Please add the suggestion based on the result and for the further studies.
