# Supplementary material for: Determinants of anemia level among reproductive-age women in 29 Sub-Saharan African countries: A multilevel mixed-effects modelling with ordered logistic regression analysis
Source: PLoS One. 2023 Nov 29;18(11):e0294992. doi: 10.1371/journal.pone.0294992 (PMC10686498; doi:10.1371/journal.pone.0294992)
Supplement: Attachment — Submitted filename: Response to Reviewers.docx [file pone.0294992.s002.docx]

**Dear Academic Editor,**

Thank you for the opportunity to revise our manuscript entitled “***Determinants of anemia level among reproductive-age women in 29 Sub-Saharan African countries: a multilevel mixed-effects modeling with ordered logistic regression analysis (ID:*** ***PONE-D-23-04658)***”. We have intensively addressed the concerns raised by the reviewers using a point-by-point response as stated below. The amendments made to the manuscript have been presented using track change in the attachment titled “Revised manuscript with track changes”.

**Responses to Editor’s Comments**

**Comment:** There are numerous studies on anemia among similar population in sub-Saharan Africa, utilizing DHS data. Please explain what distinguishes your analysis from the existing studies. Additionally, ensure that you accurately interpret your findings.

**Response:** Thank you for your important concern. As stated in the revised manuscript, we have indicated the existing gap in addition to methodological gaps as follows.

“Evidence on the level of anemia and its determinants at the SSA level is limited and most of the previous studies across this region were limited to a single country [24, 30-33] and some African countries [20, 26, 34, 35]. We have thoroughly checked and corrected the interpretation of the results.

**Responses to Reviewer-1 Comments**

**Comment:** The paper is well written and the findings and conclusions are supported by the data. The research problem of the study is clearly defined and easily understandable and the objective of the paper has been well answered and addressed. I propose the paper be accepted for publication.

**Response:** Thank you very much for accepting our manuscript in its current form.

**Responses to Reviewer-2 Comments**

The authors have addressed an important public health concerns in SSA. I would like the authors to respond to these issues I have raised.

**Comment 1:**  106 data that was collected earlier than 2010 (Central African Republic, Sudan, Nigeria Ondo State, 'Nigeria Ondo State' is not a country. Ondo State is one of the 37 state administrations in Nigeria. Please find out what DHS mean by Nigeria Ondo State and why it was conducted in isolation from the NDHS.

**Response 1:** Thank you for your important concern. We agree with your concern that Nigeria Ondo State is one of state administration in Nigeria, however in the DHS database; the dataset is available for both Nigeria and Nigeria Ondo State separately. Most importantly, data from Nigeria Ondo State was not used in the current analysis, since the survey was conducted earlier than 2010.

**Comment 2:** 113 and then appended them after managing missing observations. Please, report how you managed the missing observations.

**Response 2:** Thank you for your suggestion. We have explained this concern in the revised manuscript.

**Comment 3:** 140 First, bivariable 141 ordinal logistic regression analysis was done and proportional odds assumption was checked for 142 each explanatory variable using a post-estimation test (i.e. Brant test) [35, 36]. This procedure you have carried out is not very clear. Do you mean unadjusted ordinal logistic regression was done and Brant test was conducted for each predictor variables as post-estimation? Please make it clearer.

**Response 3:** Thank you for your concern. As you said, it is to mean, “Unadjusted ordinal logistic regression was done and Brant test was conducted for each predictor variable as post-estimation” and we have corrected it as suggested in the revised manuscript.

**Comment 4:** Secondly, I am not sure if it is a gold standard to conduct Brant test for single level would mean satisfying the proportional odds assumptions. The references you added conducted the Brant test for single level, and not multilevel. To avoid misleading your reading audience seeing this practice as the gold and acceptable standard, you may wish to include in your limitations on what you have done.

**Response 4:** Thank you for your concern. As stated, this test is descriptive and used to check whether each explanatory variable has fulfilled the proportional odds assumption i.e. it can be used with ordinal outcome variable regardless of the data structure (single level/hierarchical) since it is descriptive.

**Comment 5:** 204 final multilevel ordinal logistic regression model. It was revealed that women in a polygamous 205 union [AOR (95% CI) = 1.16(1.12, 1.21)] had a 16% greater chance of having a higher anemia 206 level than those in a monogamous union.

I have noticed that you have interpreted the results of ML ordinal logistic results as it would have been if they were binary cases. I think the interpretation should be something like this:

“It was revealed that women in a polygamous union [AOR (95% CI) = 1.16(1.12, 1.21)] had a 16% greater chance of having a higher anemia versus the combined of no anemia, mild anemia, and moderate anemia, level when compared with those in a monogamous union”.

**Response 5:** Thank you for your suggestion. We have incorporated the suggested interpretation in the revised manuscript.

**Comment 6:** 216 underweight [AOR (95% CI) = 1.21(1.10, 1.27)], had unimproved toilet [AOR (95% CI) = 1.20 217 (1.16, 1.24)], unimproved water source [AOR (95% CI) = 1.34(1.30, 1.38)],

In DHS data, wealth status was created using some variables as proxies which include improved toilet, water source, etc. In your multivariate analysis, I am not expecting you to include these variables (proxies of wealth status) simultaneously with the wealth status. Do you think it is the right thing to do?

**Response 6:** Thank you very much. We agree with your concern that these variables may be correlated. However, the selection of variables in the current study was based on the literature and the existence of multicollinearity. Most of the previous studies reported the effect of these variables (toilet facility, water source, and household wealth) on anemia, and on collinearity diagnostic, there was no evidence of multicollinearity. Due to this, we considered them in the final analysis.

**Response to Reviewer -3 Comments**

I thank you for the opportunity to read and revise the manuscript “Determinants of anemia level among reproductive-age women in Sub-Saharan African countries: a multilevel mixed-effects analysis with ordered logistic regression modeling”.

The manuscript sounds very interesting and the promising data can significantly add information on the anemia level and influencing factors in SSA. However, some parts are very hard to read, and as well as there are a consistent number of statistical issues that need to be considered.

**Requested revisions**:

**Abstract**

**Comment 1:** In method section “Data from 205,627 reproductive-age women from the recent demographic and health survey of ….” Which DHS conducted year you used please specify.

**Response 1:** Thank you very much. We have specified the survey year as suggested in the revised manuscript.

**Comment 2:** Methods used should be reflected in the abstract very well and precisely.

**Response 2:** Thank you. We have provided the details of the methods used as suggested in the revised manuscript.

**Comment 3:** Result part needs re-write and revision.

**Response 3:** Thank you. We have revised it accordingly based on the study’s objectives.

**Introduction**

**Comment 1:** Clear gap should be present other than methodological gap.

**Response 1:** Thank you for your important concern. As stated in the revised manuscript, we have indicated the existing gap in addition to methodological gaps as follows.

“Evidence on the level of anemia and its determinants at the SSA level is limited and most of the previous studies across this region were restricted to a single country [24, 30-33] and some African countries [20, 26, 34, 35].

**Comment 2:** In general the background needs extensive revision.

**Response 2:** Thank you for your suggestion. We have revised this section accordingly.

**Methods**

**Comment 1:** Please use sub-heading as “data extraction method”, I haven’t see data extraction method and where it was obtained.

**Response 1:** Thank you very much for your concern. We have provided the information regarding data extraction under the sub-heading “Data extraction and management of missing observations”. In addition, details about where data was obtained are indicated under the sub-heading “Data source”.

**Comment 2:** Line 113 “…. After managing missing observations” which missing mechanism technique you apply in this study. Please use “missing mechanism “as sub-heading explains more how you handle you missing data in your study.

**Response 2:** Thank you for your concern. As stated under the “Data extraction and management of missing observations” sub-heading, this is to indicate that women who had a missing observation on the outcome variable were dropped/excluded from the analysis.

**Comment 3:** Data cleaning procedure was not mention

**Response 3:** Thank you very much. Information regarding data cleaning was stated under the “Data management and statistical analysis” section of the revised manuscript.

**Comment 4:** For the whole method section, please try to reflect the real activities undertaken by organizations who conducted the method and data collection.

**Response 4:** Thank you. As mentioned in the method section, we have used secondary data from DHS of 29 Sub-Saharan African countries, which was collected through the collaboration of the Central Statistics Agency of each country and ICF international. Details about activities undertaken by organizations can be accessed online at:

(<https://dhsprogram.com/Methodology/index.cfm>).

**Comment 5:** The other serious problem, that requires serious attention, I have seen in this section is that, the authors did fall short of accounting for the complexity of the DHS data. The data were collected via multi-stage sampling approach with clustering and stratification. So, this data should be analyzed with this sampling process in mind. See the DHS analysis manual for better understanding. I would argue that findings without accounting for the complexity of the data are erroneous and biased towards oversampled areas and settings, and would not reflect the situation of each country.

**Response 5:** Thank you very much for your important concern. As stated in the methods section of the manuscript, we have accounted for the complexity of the sampling design by applying a multilevel modeling with an ordinal logistic regression analysis.

**Comment 6:** For each category of outcome variable cut point was not mention i.e. for not anemic, mild anemic, moderate anemic and sever anemic and cite where the cut point you found.

**Response 6:** Thank you very much for your interesting comment. We have mentioned the cut-off point for each anemia level and supported it with citations as suggested.

**Comment 7:** I haven’t seen any inclusion and exclusion criteria for this study.

**Response 7:** Thank you. We have excluded women who had a missing observation on the outcome variable (anemia level) from the study.

**Data management and statistical analysis**

**Comment 1:** How you judge the variable to have a statistically significant association with anemia level? And how you link the strength ….. Please write in this section.

**Response 1:** Thank you very much. Information about the statistical significance of the independent variables is stated under “Model building and selection” of the revised manuscript.

**Comment 2:** For the logistic regression model, you are going to use the Generalized Variance Inflation Factor (GVIF) since this model belongs to the class of generalized linear models. Results on GVIF are not presented.

**Response 2:** Thank you very much for an interesting suggestion. We have computed GVIF for all variables included in the final model and presented the result in Table 1.

**Model building and selection**

**Comment 1:** The model parameter estimation technique was not mentioned

**Response 1:** Thank you very much. The estimation of model parameters is stated under the “Model building and selection” of the revised manuscript.

**Comment 2:** What is your alternative model if the proportional odds model is failed…. I haven’t seen any other alternative ordinal model if the proportional odds model is failed….. I recommend the researcher to write other ordinal models (i.e. generalized ordered models, partial proportional odds model …) and when it applies these models in the method section.

**Response 2:** Thank you. We agree with your recommendation that other ordinal models should be considered when the proportional odds assumption is violated. However, as stated in the “Data management and statistical analysis” section of the revised manuscript, this assumption was satisfied in our analysis. Thus, we did not consider/discuss other models since the proportional odds assumption was fulfilled.

**Comment 3:** Is deviance the model selection criteria??? The model selection criteria are based on AIC and BIC values but I haven’t seen in this section. In addition testing overall model fit is not mention.

**Response 3:** Thank you very much. Deviance value (-2 * (Log Likelihood) is used as a model comparison since used a nested model (a multilevel). We have also presented other model section parameters (AIC, BIC, and LL) in Table 4 of the revised manuscript.

**Comment 4** recommend including “marginal effect” i.e. the effect of each response category across the country.

**Response 4:** Thank you for your recommendation. The effect of each variable was presented in the regression table.

**Results**

**Comment 1:** I think prevalence of anemia among reproductive-age woman should present for each country.

**Response 1:** Thank you. We have presented the prevalence of anemia for each country in Figure-1.

**Comment 2:** Result interpretation needs revision.

**Response 2:** Thank you very much. We have revised this section as suggested.

**Comment 3:** I think for this study country variation (substantial heterogeneity across each country) should reflect other than pooled prevalence.

**Response 3:** Thank you for your recommendation. We have reflected the variation in the anemia level across the included countries in addition to the pooled prevalence in the revised manuscript. In addition, details about the anemia level for each country are presented in Figure-1.

**Discussion**

**Comment 1:** Some parts in the discussion are difficult to understand. Please carefully rewrite this section considering every single set of covariates reporting both significant and non-significant results.

**Response 1:** Thank you for your suggestion. We have revised this section accordingly.

**Comment 2:** Go through the whole document and edit technical issues

**Response 2:** Thank you. We have edited the manuscript to address grammar and editorial errors.

**Conclusion**

**Comment 1:** Your conclusion should be revised and Please add the suggestion based on the result and for the further studies.

**Response 1:** Thank you for your important recommendation. We have revised this section and provided suggestions for further studies.

-------------------------------------------------------------------------------------------------------------------------------
