# Supplementary material for: Determinants of anemia level among reproductive-age women in 29 Sub-Saharan African countries: A multilevel mixed-effects modelling with ordered logistic regression analysis
Source: PLoS One. 2023 Nov 29;18(11):e0294992. doi: 10.1371/journal.pone.0294992 (PMC10686498; doi:10.1371/journal.pone.0294992)
Supplement: Attachment — Submitted filename: Response to Reviewers Comments.docx [file pone.0294992.s003.docx]

**Dear Academic Editor,**

Thank you for the opportunity to revise our manuscript entitled “***Determinants of anemia level among reproductive-age women in 29 Sub-Saharan African countries: a multilevel mixed-effects modelling with ordered logistic regression analysis (ID:*** ***PONE-D-23-04658R1)***”. We have intensively addressed the concerns raised by the reviewers using a point-by-point response as stated below. The amendments made to the manuscript have been presented using track change in the attachment titled “Revised manuscript with track changes”.

**Responses to Editor’s- Comments**

Please review your reference list to ensure that it is complete and correct. If you have cited papers that have been retracted, please include the rationale for doing so in the manuscript text, or remove these references and replace them with relevant current references. Any changes to the reference list should be mentioned in the rebuttal letter that accompanies your revised manuscript. If you need to cite a retracted article, indicate the article’s retracted status in the References list and also include a citation and full reference for the retraction notice.

**Response:** Thank you very much. We have checked all references for the stated concern and confirm that there was no retracted reference in the list.

-------------------------------------------------------------------------------------------------------------------------------

**Responses to Reviewer-2 Comments**

I am afraid, you have not advanced enough reasons for the following comments:

**Comment 1**. The Nigeria Ondo State survey is not needed in this analysis, therefore it should be presented in such a manner that it will not confuse the readers to assume that the Ondo State is one among the national surveys. If it is not needed, then remove it from your statements. It might be mis-leading others who may want to include data beyond your 2010 limit, and may assume Nigeria Ondo State is a national representative survey.

**Response:** Thank you for your recommendation. We have removed Nigeria Ondo State as suggested from the revised manuscript.

**Comment 4**. I am not satisfied with the reasons given for using the Brant test to evaluate proportionality assumptions for an ML. It is not a gold standard. The fact that others may have assumed it, as far as I know, is not the gold standard. A statement of caution should be made either at the point of usage or as a limitation. The danger is that when your paper is published as it is other researchers will begin to reference your paper as standard. If you found references that have substantiated the validity of using the Brant test for ML, you may include it in your paper.

**Response:** Thank you again for your important concern. As per the literarture, there are many tests that are used to assess the proportionality assumption in ordinal logistic regression analyses like Wald, likelihood ratio (LR), Brant, and Wolfe and Gould. As stated previously these tests are descriptive and used to check whether each explanatory variable has fulfilled the proportional odds assumption i.e. it can be used with ordinal outcome variable regardless of the data structure (single level/hierarchical). We have rechecked the assumption using likelihood ratio (LR) and Brant tests. However, we were unbale to run the other tests since there is no STATA command for the remaining tests. Thus we have acknowledged this concern in the limitation section as suggested.

**Comment 5**. You did not change anything in the interpretations as you have stated in your comment. I can't find any change.

**Response:** Thank you very much for your rimnder and sorry for the technical error. We have changed the interpretation as previously suggested in the revised manuscript.

**Comment 6**. I do not agree that you can add simultaneously variables that you have used as proxies to 'wealth status' in a multivariate model. If it is in an unadjusted model, that will be understood. Simply that the multicollinearity check could not fish it out, will not make it the right thing to do. This may affect the reproducibility of your work. If you have references that have proved that this procedure is acceptable, then include them in your paper, otherwise, let it be clear to your reader what you have done, and why you decided to do so.

**Response:** Thank you once again. As we stated in the previous reposne, we agree with your concern that these variables may be correlated. However, the selection of variables in the current study was based on the literature and the existence of multicollinearity. Previous studies have reported the effect of these variables (toilet facility, water source, and household wealth) on anemia. Moreover on the collinearity diagnostic, there was no evidence of multicollinearity, due to this, we considered them in the final analysis. Please, see the articles that have reported these vaiables below:

<https://doi.org/10.1186/s13690-021-00733-x>

<https://doi.org/10.1016/j.cegh.2021.100948>

<https://doi.org/10.1371/journal.pone.0236449>

<https://doi.org/10.3390/nu13082745>

-------------------------------------------------------------------------------------------------------------------------------
